# Left hemisphere enhancement of auditory activation in language impaired children

**DOI:** 10.1038/s41598-019-45597-y

**Published:** 2019-06-24

**Authors:** Sam van Bijnen, Salme Kärkkäinen, Päivi Helenius, Tiina Parviainen

**Affiliations:** 1 Centre for Interdisciplinary Brain Research, Department of Psychology, University of Jyväskylä, P.O. Box 35, FI-40014 Jyväskylä, Finland; 2 Department of Mathematics and Statistics, University of Jyväskylä, P.O. Box 35, FI-40014, Jyväskylä, Finland; 3 Division of Child Neurology, Helsinki University Hospital, P.O. Box 100, FI-00029, HUS Helsinki, Finland; 4 Meg Core Aalto Neuroimaging, Aalto University, P.O. Box 15100, FI-00076, AALTO Espoo, Finland

**Keywords:** Sensory processing, Neurophysiology

## Abstract

Specific language impairment (SLI) is a developmental disorder linked to deficient auditory processing. In this magnetoencephalography (MEG) study we investigated a specific prolonged auditory response (N250m) that has been reported predominantly in children and is associated with level of language skills. We recorded auditory responses evoked by sine-wave tones presented alternately to the right and left ear of 9–10-year-old children with SLI (n = 10) and children with typical language development (n = 10). Source analysis was used to isolate the N250m response in the left and right hemisphere. In children with language impairment left-hemisphere N250m responses were enhanced compared to those of controls, while no group difference was found in the right hemisphere. Consequently, language impaired children lacked the typical right-ward asymmetry that was found in control children. Furthermore, left but not right hemisphere N250m responses correlated positively with performance on a phonological processing task in the SLI group exclusively, possibly signifying a compensatory mechanism for delayed maturation of language processing. These results suggest that enhanced left-hemisphere auditory activation reflects a core neurophysiological manifestation of developmental language disorders, and emphasize the relevance of this developmentally specific activation pattern for competent language development.

## Introduction

Although the maturing brain is pre-eminently suitable to acquire language, some children have difficulties in learning to fluently speak or understand their native tongue for no apparent reason. Approximately 5% of primary school children (6–11 years) are estimated to have specific language impairment (SLI, also known as developmental language disorder, DLD^1–3^). Cognitive impairments in SLI include deficits in speech perception^4^, working memory and phonological short-term memory^5–7^. Its causes are still unknown, although it has been suggested it is a, heterogeneous, heritable neurodevelopmental disorder that can affect auditory processing^8^. Indeed, children with SLI have demonstrated altered processing of auditory information and atypical evoked brain responses to sounds^9–12^.

The sequence of brain responses to passive auditory stimulation have originally been characterized using EEG scalp recordings as a waveform with positive and negative peaks with the nomenclature focused on the order of the peaks (P1-N1-P2-N2) or latency (e.g. N100, N250). The peaks that dominate the mature and the developing auditory evoked response differ substantially. In short, whereas the adult waveform is typically dominated by the short lived P1-N1-P2 responses, the child waveform is characterized by a peak around 100 ms (referred to as P1 in EEG and P1m in MEG recordings)^13,14^ and one robust peak around 250 ms after stimulus presentation (N250/N250m or N2/N2m)^15–18^. In primary school children (~6–11 years), the emerging N1(m) overlaps in space and time with both the P1(m) and the N250(m). This complicates the isolation of the N250m and emphasizes the need to include source information to reliably separate and extract neurophysiological signatures that reflect distinct processes. When the underlying neural generators of these main components in the child waveform are modelled with equivalent current dipoles (ECDs), they reflect currents with an anterosuperior direction (P1(m)) and an inferior-posterior direction (N250(m) and N1(m))^14–16,19^.

The developmental changes in the N250(m) suggest it is an important signature of (auditory) brain maturation. The N250(m) starts to gradually decrease in amplitude around a certain age (~10–11 years) until it is no longer or barely visible in the adult waveform^16–18,20^. This decrease has been attributed to cortical reorganization, as more efficient cortical networks are established during development^17,18^. Nevertheless, it has been less intensively studied, arguably because the N1(m) is the most dominant response in adults^21^. Similarly, the P1(m) in children received more attention, possibly because it is argued to be the most dominant response in children^22^, especially during early years.

Even though the child N250(m) shows a similar source configuration as the adult N1, they most likely reflect functionally distinct processes^18,19,23^. For example, the N1 and N250 are differentially affected by inter stimulus intervals (ISIs) and thus have different refractory properties. Shortening the ISI attenuates the N1(m) while the N250(m) is enhanced or unaffected^18,24,25^. By changing the experimental design one can emphasize either component.

The buildup of N250m signal strength with shorter ISIs suggests it has a role in neural models of learning^25^. The idea that processing at this time-window reflects increased receptiveness to learn new items fits well with recent studies that have related prolonged or stronger activity in this time-window, particularly in the left hemisphere, to poorer performance on language related tasks^19,26^. This evidence suggests that left hemisphere auditory cortex activity around 250 ms plays a crucial role in processing language until more efficient cortical networks are established. Its potential role in language learning makes it especially interesting for SLI. However, to our knowledge there are no earlier studies focusing on the source activity in this time-window in children with SLI.

Earlier studies on auditory processing in SLI and dyslexia suggest deviances in P1-N1-P2 complex to simple speech and non-speech sounds^9–12,27–31^, but the results are mixed. The few studies focusing on N250 in dyslexia reported either enhanced activation in dyslexics^26,31^ or no difference to controls^32,33^.

Hemispheric differences are likely to clarify discrepancies between studies and may provide pivotal information for understanding atypical language development. Typically developing children generally show a hemispheric preference for auditory brain responses^13,14,34^, and it has been proposed that atypical auditory lateralization is the core underlying neural deficit of dyslexia^34^. Studies using EEG are, however, limited in their spatial sensitivity and less sensitive to hemispheric differences, possibly leading to a failure to consistently show a role for hemisphere-specific changes in (language) development. MEG can readily distinguish between sources in the auditory cortices of the left and right hemisphere and can utilize the components’ source information to separate functionally distinct processes that mature differently^35^. Indeed, a longitudinal MEG study of auditory evoked responses and language development in typically developing children reported a positive correlation between an increase in P1m amplitude in the left hemisphere and linguistic tests^14^. Nevertheless, the functional significance of having atypical auditory cortical responses for language development has not been established.

The aim of the present experiment was to map typical and atypical N250m responses and to study its functional significance for auditory language skills by correlating the N250m to behavioral performances. Using MEG, we compared the auditory evoked dipole source activity in the N250m time window (~250 ms post-stimulation) of children with SLI and with typical language development in response to passively listening to sine-wave tones presented alternately to the right and left ear. The use of alternating tones allowed us to look at ipsi- and contralateral stimulation and to investigate possible differences between the two hemispheres in more detail. Based on the previous literature we hypothesized stronger neural activation approximately 250 ms after auditory stimulation in the left auditory cortex of children with impaired language development compared to typically developing children. We had no hypotheses pertaining to the behavioral performances, which were used to analyze post-hoc correlations.

## Materials and Methods

### Subjects

The original source of the data reported here is a larger study by Helenius and colleagues, but only the behavioral results reported in Table 1 overlap with the original study^36^. Eleven children with SLI (mean age 9 years 8 months; age range from 106 to 127 months) and ten typical developing (TD) children (mean age 9 years 6 months; age range from 110 to 118 months) participated in that study. One child did not complete this particular passive listening task, resulting in a group of ten children with SLI (3 females) and ten TD children (3 females).

**Table 1:** Cognitive profiles of the typically developing (TD) and language impaired (SLI) children.

	TD Children	SLI children	p Value
Vocabulary^a^	11.2 (2.5)	8.5 (3.4)	ns, p = 0.062
Block design^a^	11.1 (1.9)	11 (3.2)	ns, p = 0.932
Digit span^b^	7.0 (1.2)	5.9 (1.1)	ns, p = 0.051
Pseudoword repetition^c^	11.5 (1.2)	7.7 (4.0)	p = 0.015
Sentence repetition^c^	10.0 (2.8)	5.7 (4.1)	p = 0.013
Phonological processing^c^	11.4 (2.3)	7.9 (2.5)	p = 0.004
Sentence reading^d^	13.2 (3.1)	11.1 (4.2)	ns, p = 0.221
Reading speed (min)	83.1 (35.5)	66.3 (30.4)	ns, p = 0.270
Naming speed (ms)^e^	46.6 (9.8)	47.5 (7.8)	ns, p = 0.822

^a^WISC-III standard score ^b^WISC-III raw score^39^, ^c^NEPSY standard score^40^, ^d^ALLU^41^, ^e^RAS^43^, standard deviations in parentheses, p values from t-tests (adapted from Helenius and collegagues^36^ with permission).

All participants were contacted through a larger study aiming to highlight the etiology, linguistic development and prognosis of SLI in the City of Vantaa, Finland^37,38^. The children in the SLI group had been diagnosed at the Helsinki University Central Hospital prior to school entry. All subjects were native Finnish speakers; one SLI child had a bilingual background. An informed consent was obtained from all subjects and/or their legal guardians, in agreement with the prior approval of the Helsinki and Uusimaa Ethics Committee at the Helsinki University Hospital. The experiments were approved by the Helsinki and Uusimaa Ethics committee and the methods were carried out in accordance with guidelines and regulations. The present study reports on the passive listening task not reported in the earlier articles. The behavioral results have been published before^36,37^.

### Behavioral testing and analysis

All subjects were tested on a concise neuropsychological test battery tapping non-linguistic reasoning^39^ (Block design), vocabulary^39^, verbal short-term memory and reading related skills (Table 1). In the block design test, the subject is required to copy a pattern from a figure using colored blocks, in order to assess their ability to understand complex visual information. Verbal short-term memory was tested using the digit span forward subtest^39^ and the sentence repetition tests^40^ and phonological encoding/decoding with the pseudoword repetition test (NEPSY). In these tests, the subjects have to repeat a sequence of numbers, pseudowords or complete sentences. A measure of oral reading speed was obtained from silent reading of sentences^41^ and reading aloud a narrative passage (the number of words read in 1 min). The sentence reading test (ALLU)^41^ consists of 20 trials composed of a picture that matches one of the four written sentences. The task is to identify as many correct picture-sentence pairs as possible in 2 min and the total score is the number of correctly identified sentences. Naming speed was estimated as the time to name color squares, digits^42^ (RAN) or color squares, letters and digits in a 5 × 10 matrix^43^ (RAS). Phonemic awareness was assessed using the phonological processing subtest of NEPSY^40^. The main purpose of the behavioral testing was to provide cognitive profiles for both groups (Table 1), not to diagnose SLI, as the SLI subjects had been diagnosed earlier. However, in order to study the functional significance of the auditory response, scores were also used to analyze post-hoc correlations between the behavioral tests that showed differences between groups (p < 0.10, Table 1) and the neural responses of interest (i.e. N250m). To do this, we used Kendall’s tau non-parametric correlation because it is more appropriate for small data sets and/or when participants have the same scores, as in the current study^44^. We controlled the false discovery rate by using the Benjamini-Hochberg procedure^45^. Hannus and colleagues^37^ provide interpretation of the different cognitive profiles in an earlier paper. In short, we expect the different p-values of the tests to reflect their sensitivity and specificity to diagnose SLI in Finnish children.

### Stimuli and MEG recordings

The stimulus was created using Sound Edit (Macromedia, San Francisco, CA, USA) and consisted of a monaural 50-ms (15-ms rise/fall time) 1-kHz sine wave tone, 65 dB HL. Stimuli were presented alternately to the left and right ear in order to probe ipsi- and contralateral auditory pathways in each hemisphere. Inter-stimulus interval (ISI) varied randomly between 0.8- and 1.2-s.

During the measurement, the child and one accompanying adult were seated in a magnetically shielded room and instructed to avoid excessive head movements. Stimuli were controlled with the Presentation program (Neurobehavioral Systems Inc., San Francisco, CA, USA) running on a PC and delivered to the subject through plastic tubes and earpieces. The children were asked to ignore the tones and they watched a silent cartoon during the whole recording.

The auditory cortical responses were recorded using a 306-channel whole-head system (Vectorview™, Elekta Neuromag Oy, Helsinki, Finland). This system measures magnetic field strength at 102 locations over the scalp; two orthogonally oriented planar gradiometers and one magnetometer at each location. Prior to the measurement, four head-position indicator (HPI) coils were attached to the participant’s scalp. HPI coils were digitized with a 3-D digitizer in order to determine their location in relation to three anatomical landmarks; preauricular points and nasion. At the start of the measurement, HPI coils locations with respect to the MEG helmet were measured. Finally, eye blinks and movements were monitored by placing electro-oculogram (EOG) electrodes directly below and above the right eye and on the outer canthi of each eye.

### MEG analysis

The MEG signals were bandpass filtered at 0.1–200 Hz and sampled at 600 Hz. The raw data were processed using the spatio-temporal signal space separation method^46^. Offline, responses were averaged from −0.2 to 0.8 s relative to stimulus onset. Epochs contaminated by vertical or horizontal eye movements were rejected. To minimize the effect of heartbeat artifacts the MEG signals were offline averaged with respect to the heart signal and principal component analysis was used over this average to project out the resulting magnetic field component^47^. Finally, the data were manually checked to exclude epochs with major artifacts. On average 107 artifact-free averages were collected in the TD group and 111 in the SLI group.

The active source areas were modelled from the averaged data using equivalent current dipoles^48^ (ECD). Averages were filtered with a 40 Hz lowpass filter and baseline corrected (−0.2 to 0 s). Xfit software was used to estimate the localization of the current sources (Elekta, Oy, Helsinki, Finland). In each subject, the same 20 planar sensor pairs were selected in each hemisphere that best covered the dipolar field pattern. To identify the cortical response around 250 ms after auditory stimulation, ECDs were accepted when (i) in the time window of interest (175–325 ms), (ii) they had a goodness-of-fit value of >80% and (iii) they had a predominantly inferior-posterior direction. These criteria were based on the pattern of activation that is most reliably repeatable for this specific time-window. ECD locations and orientations were fixed, while their amplitudes were allowed to vary. In each subject, the magnetic field patterns were visually inspected to identify local dipolar fields in each stimulus condition (i.e. ear and hemisphere). From the resulting four ECDs one was selected in each hemisphere that best fit the data in all conditions. As individual MR images of the subjects were not available, a spherical volume conductor model was used with the default center defined as the origin (0, 0, 40). Dipole moment amplitudes were defined as the average of the peak (175ms–325ms). Data points around the peak were included as long as they exceeded two standard deviations above the mean activation of the whole epoch.

### Statistical model and analysis

The data were analyzed using R^49^ and the packages *lme4* and *pbkrtest*^50,51^. The amplitude values of each factor: ear (2) and hemi (2) were extracted for each participant in each group (2), resulting in four amplitude values for each participant.

In order to assess the effect of impaired language development on auditory evoked source activity we used a linear mixed model (LMM) or, more specifically, a random intercept model^44,52,53^. A random intercept model is, in our case, more suitable than ANOVAs because it resolves the non-independence of multiple responses from the same subject by assuming a different baseline value for each subject (i.e. random intercept). In addition, it has more opportunities to control for possible problems that may arise due to our small sample size (i.e. power and type 1 error rate). LMM has fewer assumptions compared to ANOVAs^54^, violations of which affect the type 1 error rate and power of ANOVA F tests^55^.

In the estimation of the best model for the covariance structure (compound symmetry), we used a backward method with the maximum likelihood (ML) approach. The significance test to be used was the chi²-test based on the likelihood ratio test (LRT) backward selection heuristic of two nested models to compare the models. For small sample sizes, this approach was reported to be more conservative compared to the Akaike information criterion (AIC), maintaining the type 1 error rate of a maximum model^56^, while increasing the power substantially^57^. The final model is a collection of fixed and random effects. Here, we calculated it with the restricted maximum likelihood (REML) approach to reduce the bias of the estimators of variances of the random intercept and the residual. The REML is less affected by a small sample size and has consistently shown lower type 1 error rates^58,59^.

In the diagnostics part of the model, we first confirmed the normality of residuals to be valid using a qq-plot and a scatter plot for groups. Furthermore, we established the normality of random intercepts utilizing a qq-plot. Using the final model, we defined contrasts for separate sets of regression coefficients. For testing if the contrast is zero, we used the Kenward-Roger (KR) approximation for F-test by Halekoh and Højsgaard^51^, as this method produced acceptable type 1 error rates even for smaller samples^59,60^. In the contrast calculations from KRmodcomp, we obtained the numerator d = 1. Then, F statistics is the squared t statistics, and for simplicity, contrasts are reported by using t-statistics, dfs and p-values.

## Results

### Source analysis

**Figure 1:**
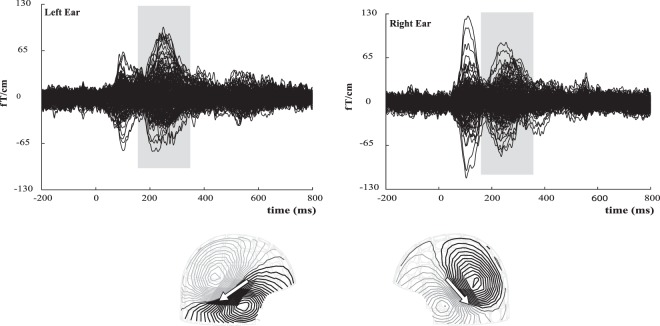
Butterfly plot of signals recorded by gradiometer sensors to left and right ear stimulation of one participant (top). ECD’s were selected in the time-window of interest (window). The bottom figure shows the typical field distribution and dipole orientation (arrows).

In 90% of the participants (18/20) we were able to select at least one dipole in each hemisphere in the time-window of interest. In the other two (one in SLI group and one in TD group) we were unable to find dipoles meeting our criteria and thus they were excluded from further analysis. In Fig. 1, the gradiometer butterfly plots of the auditory evoked fields of one participant are shown; the time window used for source localization is marked by a window. The corresponding field distributions and dipole orientations are depicted on the bottom of Fig. 1.

**Figure 2:**
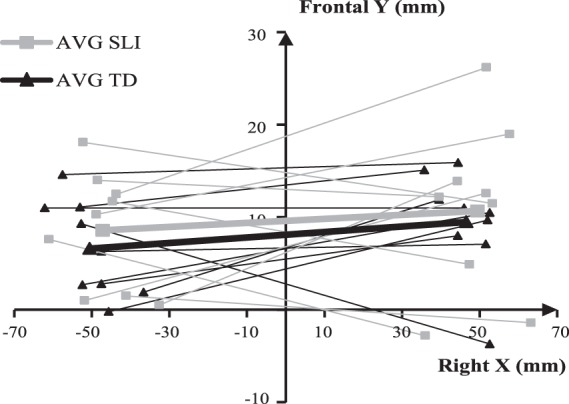
Dipole x and y coordinates in axial plane of each participant (thin lines) in SLI (grey) and TD group (black) as well as their averages (thick lines).

**Figure 3:**
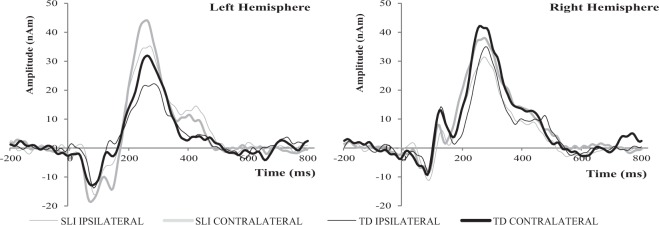
Grand average time-course of activation of the dipolar sources in the left and right hemisphere plotted separately for contralateral (thick lines) and ipsilateral (thin lines) responses for SLI (grey) and TD (black) group.

Figure 2 shows the location of the selected dipoles (x, y coordinates in axial plane) of each individual and the grand average location of the two groups. Figure 3 depicts the resulting grand average waveforms. There were no significant differences between the groups on any of the (x, y, z) coordinates (*p* ≥ 0.28).

### Modelling

In the first most inclusive model we had all variables (group, ear, and hemi), their pairwise interactions and a three-way interaction. Using a cut-off of α = 0.05, we dropped first the three-wise interaction group*ear*hemi, chi²(1) = 0.085, *p* = 0.78, and second the pairwise interaction group*ear, chi²(1) = 0.006, *p* = 0.94. Furthermore, the equality of variance in SLI and TD group was checked and could be assumed, chi²(1) = 1.263, *p* > 0.26. The final random intercept model was calculated using the restricted maximum likelihood (Tables 2 and 3). In Table 2, the estimates, standard errors and their ratios (t-values) are shown. Estimates and confidence intervals of the random effects are shown in Table 3.

**Table 2:** Fixed effects of the model: estimate (standard error(s.e.)), degrees of freedom, t-value and p-value.

	Est. Value (s.e.)	DF	t-value	p-value
(Intercept)	33.12 (4.28)	50	7.730	0.000
Group^a^	−0.45 (5.52)	16	−0.082	0.936
Ear^a^	8.34 (3.52)	50	2.368	0.022
Hemi^a^	−1.49 (4.31)	50	−0.346	0.731
Group × Hemi	13.19 (4.98)	50	2.647	0.011
Ear × Hemi	−13.71 (4.98)	50	−2.752	0.008

^a^Baseline for group: TD, for ear: Right and for hemi: Right.

**Table 3:** Approximate 95% confidence intervals for the standard deviation of random effects.

	Lower	Est.	Upper
Intercept	5.64	9.03	14.44
Residual	8.69	10.57	12.86

### Effect of ipsilateral vs. contralateral stimulation

**Figure 4:**
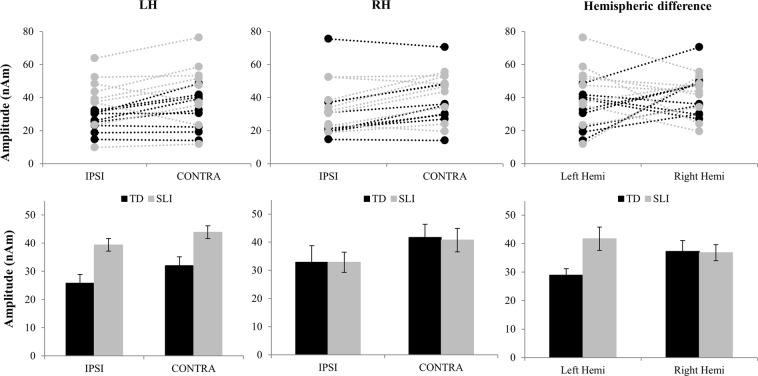
Individual (top) and averaged (bottom) strength of activation in the left hemisphere (LH; left) and right hemisphere (RH; middle) in response to ipsi- and contralateral auditory stimulation of children with SLI (grey) and typical language development (black). Hemispheric differences (right) are plotted as the difference in activation strength to contralateral stimulation (i.e. right ear for left hemisphere and vice versa). Whiskers in the bottom figures represent the standard error of the mean (SEM).

The mixed effects model revealed an interaction between ear and hemi, *t*(50) = −2.752, *p* = 0.008. Generally, contralateral stimulation showed greater amplitudes compared to ipsilateral stimulation. In the right hemisphere this estimated difference (ED) was clearer (ED = 8.34, SE = 3.52) and significant *t*(50) = 2.367, *p* = 0.022. In the left hemisphere this difference was smaller (ED = −5.37, SE = 3.52) and not significant *t*(50) = 1.524, *p* = 0.134. Figure 4 depicts the individual (top) and averaged (bottom) strength of activation resulting from ipsi- and contralateral stimulation in the left (left) and right (middle) hemisphere for both groups. The ipsi-contralateral effect did not seem to differ between groups since neither the three-way interaction (ear*hemi*group) nor the two-way interaction (ear*group) were significant (chi²(1) = 0.085, *p* = 0.771 and chi²(1) = 0.006, *p* = 0.937, respectively).

### Group differences in the two hemispheres

The mixed effects model revealed a significant interaction between group and hemisphere, *t*(50) = 2.648, *p* = 0.011 (Table 1). This effect was limited to the left hemisphere as the difference between groups (ED = −12.74, SE = 5.52) was significant in this hemisphere for both the ipsi- and contralateral stimulation, *t*(24.70) = 2.306, *p* = 0.03. In contrast, the difference between groups in the right hemisphere was negligible (ED = 0.45, SE = 5.52), *t*(24.70) = 0.082, *p* = 0.935. Figure 4 (left vs. middle) depicts the plots corresponding to this difference. Finally, TD children showed significantly higher amplitudes in the right compared to the left hemisphere (ED = 8.35, SE = 3.52, *t*(50) = 2.370, *p* = 0.022), indicating a cortical asymmetry in this group (Fig. 4; right). Children in the SLI group show the opposite pattern with stronger activation in the left hemisphere compared to the right hemisphere (ED = −4.84, SE = 3.52), but this difference was not significant *t*(50) = −1.374, *p* = 0.176.

### Correlation between behavioral skills and brain responses

**Figure 5:**
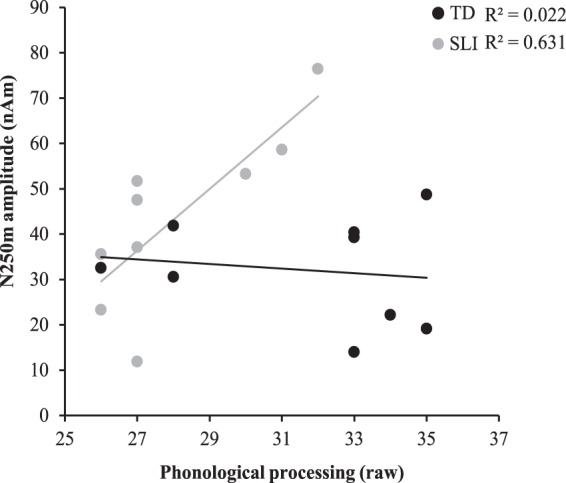
Scatterplot representing the correlation between phonological processing (raw score) and N250m amplitude in the left hemisphere, to contralateral stimulation, for the SLI (grey) and TD (black) group.

Post-hoc correlations were performed between the amplitude of the N250m to contralateral stimulation and the behavioral measures for each group. Data were checked for outliers, and none were found (all individual values < 1.8 SD). In the TD group, no significant correlations were found. However, in the SLI group, we found a significant positive correlation between phonological processing scores and N250m amplitude in the left hemisphere τ_b_ = 0.774, p = 0.006, but not the right hemisphere τ_b_ = 0.278, p = 0.321. In the SLI group, those with higher N250m amplitudes in the left hemisphere performed better on the phonological processing task (Fig. 5). When corrected for the other behavioral tests that showed differences between groups (i.e. vocabulary, digit span, pseudoword repetition and sentence repetition), the corrected p-value was 0.03.

## Discussion

In this study we assessed typical and atypical variation in the N250m response and examined its functional significance for language processing. As was hypothesized, auditory processing in the cortical time-window of the N250m was altered in children with impaired language development and this alteration was limited to the left hemisphere; N250m dipole moment in the left hemisphere was stronger in SLI children. In our view these findings illustrate the association between maturation of the auditory cortex in the left hemisphere and language development, with relevance for neurodevelopmental disorders.

Our results provide further support for the hypothesis that stronger or more sustained activation in the cortical timing of the N250(m)^31^, especially in the left hemisphere^19,26^, is indicative of less developed language skills. This cortical response is observed to be specific to the developing brain^16,17,19^ and weaker neural activation in this time window has been related to better reading skills in typical developing children^19,26,31^. Indeed, the decrease in amplitude of the N250(m) (and increase in N1(m)) have been speculated to reflect more automatized auditory processing^19,61^. Paradoxically, in our clinical group, higher N250m amplitudes in the left hemisphere were related to better performance on a phonological processing task; a core deficit in SLI and a crucial component in learning to read^62,63^. Presumably, children with SLI rely more strongly on neural sources in the left hemisphere as a possible compensatory mechanism for delayed maturation of language processing. However, this correlation should be interpreted with care, as correlations typically only stabilize at considerably larger sample sizes^64^. Therefore, we do not expect the current correlation coefficient to accurately represent the true value in the SLI population and acknowledge that this might be an over-estimation of the effect size^65^ or a type-1 error. Nevertheless, our claim is substantiated by an EEG study that identified an enhanced N250 response as a compensatory mechanism for phonological processing deficits in dyslexic children but not in typical developing children^31^.

The simplest account of our data is an enhanced auditory brain response in the left hemisphere of children with SLI. Several studies have already observed the relationship between language skills and auditory evoked responses in left hemisphere^14,36,66^ and some have focused on the N250(m)^19,26^. However, the source activity of the N250m has not been contrasted between children with typical and impaired language development. By using MEG ECD source modelling techniques, we were able to show hemisphere-specific alterations (i.e. increase in left hemisphere exclusively) in the auditory evoked responses of children with impaired language development. In our view, this illustrates the different role of the two hemispheres in developmental language disorders and emphasizes the need to include spatial information to properly distinguish between activation patterns possibly varying in time and between hemispheres. For estimating the detailed location of activation in the two hemispheres, information on individual brain anatomy should be used, which was not available in the present study. Importantly, group differences in source strength could not be explained by differences in dipole locations.

Although it is not possible to draw strong conclusions on hemispheric asymmetry based on our data, given the recent debate on the role of lateralization and asymmetry in developmental language disorders^67–70^, we will discuss findings we think are relevant to this discussion. Furthermore, we will speculate on how the interaction between hemispheres could be affected by developmental language disorders.

We used monaural stimulation in order to probe ipsi- and contralateral pathways, allowing us to investigate hemispheric differences (left vs right), laterality effects (ipsi vs contra) and their interactions. The data showed a contralaterality effect in both groups; a greater amplitude in the hemisphere contralateral to the stimulated ear compared to the ipsilateral hemisphere. Typical developing children showed overall higher amplitudes in the right compared to the left hemisphere. Both results are in good agreement with previous literature on hemispheric asymmetry in pure tone processing and contralaterality effects in children^13,71^ and adults^13,72–75^. In the present study however, children with impaired language development showed an opposite, but not statistically significant, asymmetry pattern, indicating a lack of typical asymmetry similar to what was found in dyslexic children^34^.

Given that speech vs. nonspeech processing typically reflect opposite asymmetry patterns (i.e. leftward vs. rightward respectively), it is important to distinguish between studies looking at auditory and language lateralization. In addition to opposite asymmetry patterns of speech and nonspeech processing, the theory of asymmetric sampling in time (AST) proposes that cerebral asymmetries relate more to the temporal features of auditory information. In this view, the right hemisphere samples slow (syllabic) rate auditory input (~3–7 Hz) and the left hemisphere fast (phonemic) rate auditory input (~12–50 Hz)^76–78^. For certain language processes (e.g. phonological processing), both temporal features must be integrated. This dynamic nature of cerebral asymmetry needs to be considered when discussing asymmetries and hemispheric differences in relation to language and auditory processing, and make it likely interhemispheric connections play a crucial role.

In addition to functional hemispheric differences, anatomical hemispheric differences might also explain the differences between our two groups. Indeed, studies reporting white and grey matter structural differences in children with developmental language disorder are numerous^79–81^. However, of special interest for M/EEG studies is a report that demonstrated a more convoluted auditory cortex produces stronger cancelation effects resulting in lower measured EEG and MEG signal^82^. The authors argued that the left hemisphere is typically more convoluted resulting in the rightward bias in pure-tone processing. In the present study, the enhanced auditory responses in the left hemisphere of the SLI group could be explained by a less convoluted left auditory cortex, or more focal cortical activity in the left hemisphere compared to controls^82^. Importantly however, a recent study investigating the neuroanatomical basis of developmental dyslexia identified an atypical sulcal pattern with more convolutions in left hemispheric perisylvian regions compared to controls as a biomarker of dyslexia^83^. Assuming this result can be extrapolated to our subjects, one would expect lower amplitudes in the left hemisphere in the SLI group. Future studies combining neuroanatomical and functional (MEG) data are needed to clarify laterality of auditory and language processing in developmental language disorders.

Even though it appears inevitable that an abnormal neural activity pattern in the left hemisphere disrupts the cerebral asymmetry of language processes, the question remains whether interhemispheric auditory connections are affected or that it only reflects the primary dysfunction in the left hemisphere. Due to the nature of the present study and the complexity of the auditory system, we cannot conclusively say whether this is the case. Based on our results, it is tempting to conclude that auditory pathway interactions are unaffected by impaired language development, as differences in the right hemisphere were negligible. It should be noted however, that during monaural stimulation, there is no competition between both ears. Others have argued that the stronger the competition between the ears (e.g. in a dichotic listening task), the stronger the interactions between the auditory pathways^84^.

To examine interaural interaction in developmental language disorders the ‘frequency tagging’ method can be used. With this method, auditory input to each ear is ‘tagged’ with amplitude modulations of different frequencies that can later be decoded from the cortical responses. This has proven a useful tool to evaluate the central auditory pathways in more detail^85^. Indeed, one study utilizing this method observed weaker ipsilateral suppression (a measure of interaural interaction) in dyslexics depending on the strength of ROBO1 expression (a known dyslexia gene)^86^. The authors demonstrated that the weaker this gene-expression in dyslexic individuals, the weaker the interhemispheric interaction. Interestingly, this gene is also suggested to be involved in neuronal migration underlying brain lateralization in healthy subjects with a specific function in supporting a short-term buffer for arbitrary phonological strings^87^. These results indicate that impaired language development is associated with weaker interaction between auditory pathways which may be especially detrimental for phonological processing.

Two issues regarding the increased N250m response amplitude and atypical hemispheric balance still require clarification. First, this study’s design was not well suited to determine whether they are a cause, correlate or consequence of developmental language disorder. Similar to many comparable studies, not all our SLI children show an increased N250m and atypical hemispheric balance. Thus, atypical hemispheric balance (or indeed increased N250m) should not be seen as a critical cause of SLI. We suggest it is more likely a consequence, as we argue that the increased N250m can (partly) compensate for the language deficit. It is also possible the processing differences in the left hemisphere causes problems in language-related functions or that the auditory and language deficits are both markers of an underlying neurodevelopmental disorder^9^.

Second, we are left with an apparent dichotomy where the N250m is suggested to be both indicative of poorer (in TD group) and superior (in SLI group) language skills. We do not consider it an impossibility that processing in this time-window is both an indicator of language or auditory development as well as a useful tool for the developing brain. The fact that this neural process is present in most children suggests it is beneficial for development, the fact that in adults it typically is not, suggests the brain develops a more efficient way of processing auditory stimuli. We surmise that neural processing in this time-window is exceptionally flexible, which should be a useful tool, and indeed a necessity, in the learning environment of the child brain.

This study’s main limitation is its sample size. Small sample sized studies raised considerable debate^65,88–92^ and we agree that they deserve additional scrutiny. We strived for maximal power by using methods that have specific advantages concerning type 1 error rates and statistical power with small sample size studies, namely; (i) a statistical model with fewer assumptions (LMM) (ii) model selection (LRT backward heuristic), (iii) model fit (REML), and (iv) evaluating significance (KR approximation). Furthermore, several authors have defended small-N designs, mainly for its inferential validity^88,90–93^. Nevertheless, we caution against taking our findings, especially the correlation, at face value.

To conclude, we provide evidence that neural activation at ~250 ms is functionally meaningful for the integrity of language skills and substantiate the claim that enhanced left-hemisphere auditory activation reflects a core neurophysiological manifestation of developmental language disorders. We found significantly stronger activation in the left hemisphere of the SLI group, as compared to controls, that unmistakably differed in language skills. We suggest this might reflect a compensatory mechanism for language processes. The effect was isolated to the language dominant left hemisphere and is thus in agreement with other studies associating altered neural responses in the left hemisphere to language skills and impaired language development.

## Data Availability

The dataset analyzed during the current study are not publicly available due to legal restrictions but are available from the research group on reasonable request.
